# The Uses and Abuses of Clinical Records

**Published:** 1889-07

**Authors:** Edward Cranch

**Affiliations:** Erie, Pa.; I. H. A., Bureau of Homœopathics


### THE USES AND ABUSES OF CLINICAL RECORDS.

Edward Cranch, M. D., Erie, Pa.

[I. H. A., Bureau of Homœopathics.]

Hahnemann, in his *Materia Medica Pura*, Vol. I, preface, gives, in response to requests for his mode of cure, a warning and an example, the warning being that no satisfactory deductions can be drawn from one case as to how to treat another, except as to the method of study employed, since each case cured shows only that that particular case was so cured.

Then he proceeds kindly to record two cases, with their respective groups of symptoms, and illustrates his mode of arriving at the remedy, which involves a nearly perfect knowledge of the materia medica, combined with a faculty for isolating, noting, and comparing symptoms that we should all study to acquire.

For completeness of the present subject, "The uses and abuses of Clinical Records," a classification of such records may be made, and the most interesting class studied most closely.

First, then, we may divide all clinical records into three classes—viz.: business records, hospital records, and journal records.

"Business" records should cover *every case* prescribed for, and note the remedy, the dose, and repetition, the adjunctive rules for diet, etc., if any, leaving the rest to memory, though, if there be room, a hint of the chief conditions present will not be out of place. Such records will be of great value in retaining patients who return for that which previously helped, and will give information much needed; although, owing to the imperfections of the human mind, it often happens that the very remedy that does the most brilliant work will escape record!

"Hospital" records should be such as every hospital should keep, detailing every phase of the cases that can possibly be obtained, and from such records statistics of treatment of similar groups of symptoms, sometimes conveniently called diseases, can be elaborated.

"Journal" cases: cull out from private or hospital practice such cases or groups of symptoms as are of special interest, either detailing them at large, with comments and comparisons, or noting them more briefly as "verifications" or "clinical effects" of this or that drug; then they furnish notes for our repertories and materia medica, and are, when reliable, the best material for study that we can have.

"Journal" cases should always be written with a view to their future usefulness in study, not merely for applause or wonder; and they should be carefully divested of all extraneous matter, yet including sufficient vividness of personal description and anecdote to fix them in the mind.

Hahnemann's two cured cases already alluded to belong to and exemplify the latter class of "Journal" records, and are chiefly useful for what they imply, rather than for what they directly teach.

Coming from the master, they show his wonderful knowledge of the materia medica, so largely his own creation, and his knowledge of what his remedies could *not* do, as well as of what they did. He first states, without a hint of what he *thought* or might have thought about *Pathology*, the exact symptoms that he observed, recorded singly, without apparent order or connection, concluding with the remark, "no other abnormal symptoms." Then he gives remarks on each symptom, giving the nearest related remedies in each case, and showing that only *one* has the needed combination, excluding all others in a masterly way that shows his complete familiarity with repertorial work, especially in the valuable field of concordances and concomitants.

In short, he knew how to use his materia medica. He says, "In looking out a remedy, it is sufficient to note the drugs producing the first symptom, *recollecting* the *conditions* in which the symptom is produced. This same proceeding is followed with each of the other symptoms, and that drug which contains the most striking and characteristic symptoms of the group is the remedy."

He goes on to advise young physicians to *prove remedies* on themselves; and no physician should attempt to record a group of observed symptoms until he has acquired some experimental knowledge in his own person of what a group of symptoms is. All other knowledge is faith without works, and is dead for use.

The physician who attempts to practice without having made at least a few provings is like the performer who has never composed a single piece of music; he may copy the work of others, in a fashion, but cannot do anything in new fields, or in the advancement of his art.

It is a fallacy that is sometimes taught that the best way to study the materia medica is to study out actual cases of sickness; it is true that so the knowledge of it is *fixed*, but to best learn how to use the materia medica, one should edit a few chapters therein, either as re-provings, or as new investigation for which there is always room; next, one must *study* some repertory, and be able to find a drug-picture by its aid; then, having learned what symptoms really mean, he can record and compare them better.

Given complete clinical records, in acceptable journal form, how can we use them best?

First, we can take down our text-books and compare the recorded symptoms, marking the old with renewed confidence, and setting the new in the margin for future verification.

Next, we can annotate our repertories to correspond; and, lastly, if the reporter has given the name of the disease, we can set that information by itself with a view to forming what Bell, Minton, Perkins, King, Lee, W. J. Guernsey, and others have begun—special repertories for each specialized disease or pathological form. Or we can *abuse* this information by the writing of such hand-books as Lilienthal, Johnson, and Jahr have given us, wherein diseases are named first, and *conditions* subordinated thereto; good stepping-stones for weak-kneed pathologists, but poor dependences for the Hahnemannian prescriber, who wants only complete "symptom-lists" in which he can find and compare each condition as it arises, without the uncertain task of translating it into pathology and back again. In short, the Hahnemannian most needs an accurate and copious materia medica, and its accompanying repertories, general and special; and to the improvement of these and their living useful study his use of clinical records will always contribute, and all short cuts that would say "Dr. L. or Dr. D. gave this or that remedy in a case like mine, I will give the same," he will view with suspicion, as tending to careless observation.

Cases that record the effects of single remedies and single doses are, of course, to be preferred, and such modes of prescribing, in the single remedy, the single dose, and the recorded case will stimulate the most careful observation, which all other methods will impair, and finally destroy, till it will be found that all power of accurate observation and useful study is gone forever.

